# Defibrillation failure with an electrical short circuit caused by internal insulation breach

**DOI:** 10.1016/j.hrcr.2021.04.009

**Published:** 2021-04-29

**Authors:** Takuo Tsurugi, Junjiroh Koyama, Kazuhisa Kodama, Hiroshi Nakajima, Tomohiro Sakamoto, Ken Okumura

**Affiliations:** ∗ Saiseikai Kumamoto Hospital, Kumamoto, Japan; † Chiba Nishi General Hospital, Chiba, Japan

**Keywords:** Defibrillation failure, Riata ST Optim lead, Short circuit, Silent lead malfunction, Ventricular fibrillation

## Introduction

St. Jude Medical (now Abbott) released the Riata ST Optim lead in 2006 as one of the Durata family of defibrillator leads. These leads were coated with silicone polyurethane copolymer insulation material (Optim) on Riata and Riata ST leads. In 2011, Riata and Riata ST leads were designated to be a class I recall by the United States Food and Drug Administration (FDA) because of lead insulation failure. Since the Riata ST Optim lead increased the insulation thickness by 50% after coating with 0.09-mm Optim,^1^ the rate of conductor externalization was significantly decreased, from 19%–28% in Riata and Riata ST leads^2^ to <1% in Riata ST Optim and Durata leads.^3^ As of 2020, Riata Optim leads had been implanted in >19,000 patients in the United States and the 10-year survival rate is approximately 92%, according to an Abbott product performance report.^4^ However, in contrast to improvement of conductor cable externalization, concerns of internal insulation breach, a possible cause of silent lead malfunction or defibrillation failure, remain to be determined. Herein we report a case of defibrillation failure with a lack of effective shock delivery owing to low defibrillation lead resistance (<20 Ω) in a Riata ST Optim lead, which was not revealed by routine periodic interrogation.

## Case report

A 44-year-old woman with cardiac sarcoidosis and sustained ventricular tachycardia (VT) had an implantable cardioverter/defibrillator (ICD) (Virtuso DR; Medtronic Inc, Minneapolis, MN) with Riata ST Optim dual-coil defibrillation and CapSureFix atrial leads (Medtronic Inc) implanted in 2008. Defibrillation threshold testing (DFT) was unsuccessful because induced VT or ventricular fibrillation (VF) was not sustained. Between 2008 and 2013, an estimated 10 VT/VF events were successfully terminated by 35 J shocks. The ICD generator was replaced owing to battery depletion by an Evera XT (Medtronic Inc) in 2013. Subsequently, 2 more VTs were successfully terminated by 35 J. She then developed dyspnea on effort coincident with an ejection fraction reduction to 20% and left bundle branch block, prompting the ICD to be upgraded to cardiac resynchronization therapy with a defibrillator (CRT-D) using Viva Quad XT (Medtronic Inc) and a left ventricular lead (Attain Performa 4598; Medtronic Inc) in 2015. At that upgrade procedure, DFT was not performed; however, full routine lead testing was completely satisfactory. Fluoroscopic observation revealed no conductor externalization of the Riata ST Optim lead. The patient had a good response to CRT-D and no VT episodes were documented after the upgrade.

**Figure 1:**
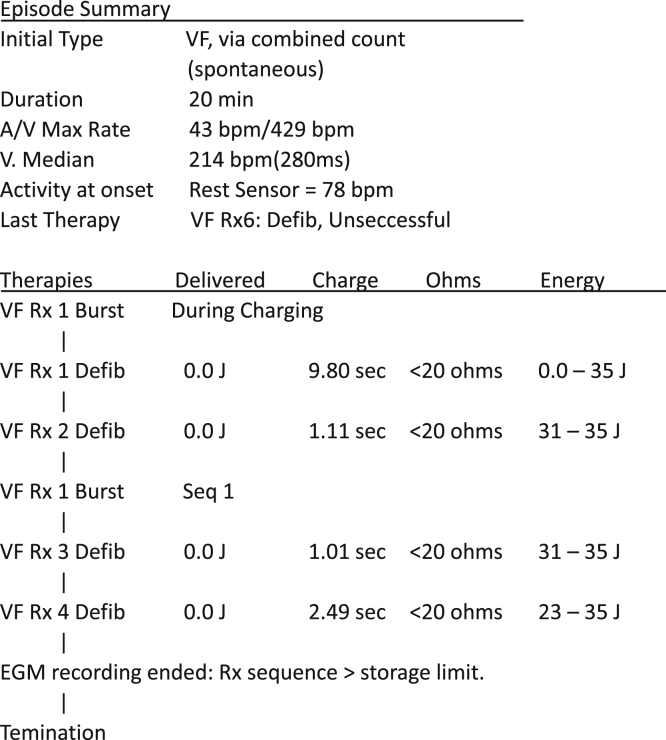
Episode summary revealed that ventricular fibrillation (VF) detection occurred following the charge of capacitor to 35 J, but a 0.0 J shock was delivered owing to the excessive current protection system.

**Figure 2:**
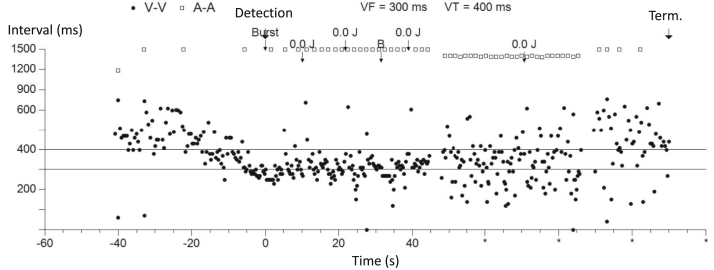
Ventricular fibrillation (VF) could not be terminated by a 0.0 J shock and burst pacing (*black arrows*), which continued for 20 minutes.

In September 2019, her family noticed that she was suddenly moaning and breathing abnormally at 2 AM. She was immediately transported by ambulance to the hospital, but cardiopulmonary resuscitation failed. Device interrogation revealed 6 VF events, for each of which shock therapy was initiated (Figure 1). The therapy was interrupted each time because of the low defibrillation lead resistance (<20 Ω), thus resulting in termination of the therapy algorithm (Figure 2). We were not permitted to remove the affected defibrillation lead for investigation. The extracted CRT-D generator revealed no arc marking around the device can. On the day before the event, remote routine monitoring data showed that right ventricular (RV) and superior vena cava (SVC) shock impedances were 38 Ω and 68 Ω, respectively, which had been nearly constant for at least 8 years.

## Discussion

There have been many adverse events reported in ICD leads, among which failure of VT/VF therapy is the most critical clinical event. Short circuit of the defibrillation lead is known as one of the mechanisms underlying failure. Previously, we described the various mechanisms and clinical presentations of electrical short circuits.^5^ The present case was similar to case 1 in our previous report. There was no arcing mark on the generator can and the device was shown to initiate therapy by delivering high-energy shocks. The excessive current, however, invoked the protection mechanism, which resulted in termination of the delivery. The treatment log in the device revealed an unsuccessful time course. The short circuit likely occurred between the SVC and RV conductor coils. In our previous review,^5^ we suggested that the SVC coil should be excluded from the defibrillation system to avoid short circuit–related failure. Unfortunately, in the present patient the device had not been programmed in this way. It should be kept in mind that this potential short circuit is extremely difficult to detect before it occurs.

### Riata ST Optim Lead

To the best of our knowledge, this is the first report of unsuccessful defibrillation in a Riata ST Optim lead owing to aborted shock delivery as a result of a decrease in impedance. Recently, Hauser and colleagues^6^ reported lead failure and adverse clinical events of Durata, in which lead construction is similar to that of the Riata ST Optim, from the 2008–2018 FDA Manufacturer and User Facility Device Experience (MAUDE) database. Of Durata lead failures, 93% were associated with insulation failure, which was at a significantly higher rate than the Sprint Quattro Secure (16%) and Endotak Reliance leads (38%). Moreover, Durata internal insulation breaches contributed to 11 failures of VT/VF termination, and all cases were caused by a lead short circuit. Ten of the 11 lead short circuits occurred between the SVC and RV coils. Since the Durata and Riata ST Optim leads had no coating with Optim under the SVC shocking coil, a distal RV shocking coil could abrade through the inner silicone from inside to outside and ethylene tetrafluoroethylene would then be damaged, allowing that coil to shortcut to the SVC shocking coil.

### How to avoid a short circuit?

The most important step in protecting patients from a short circuit is to recognize the risks inherent in the dual-coil system,^5^ Riata or Durata leads,^1^^,^^6^ and aged defibrillation leads.^7^ Indeed, there are 3 possible approaches to prevent defibrillation failure. First, removing the SVC coil from the defibrillation circuit is effective against insulation breach between the SVC and RV coils. This strategy was recommended previously.^8^ What is of most concern from reprogramming the SVC coil off is attenuation of the DFT threshold; however, a meta-analysis revealed that the single coil is not inferior to the dual coil with respect to prognosis and the initial defibrillation success rate.^9^ Reprogramming to a single-coil system does not necessarily increase the defibrillation threshold, except for those patients who selected a dual coil because of DFT problems with a single coil. Although the DFT test could confirm the integrity of the single-coil setting, it is important to recognize that the DFT test itself also has risks.^10^

Second, the automatic shocking-vector adjustment algorithm (Dynamic Tx) is also useful.^11^ In 2013, the FDA approved this algorithm, which has been available in the latest models of the Abbott ICD and CRT-D. When a short circuit occurs and an overcurrent (>60 A) is detected, Dynamic Tx automatically changes the vector configuration from “RV to SVC/Can” to “RV to Can,” followed by the “RV to SVC” setting until a high-voltage shock is completely delivered. Using the vector of “RV to Can” or “RV to SVC” could overcome an insulation failure not only between the SVC and RV coils, but also in the RV coil to Can. Unfortunately, an important limitation of Dynamic Tx is that operation of Dynamic Tx requires the defibrillation system to use a single-coil system, even though some patients have used a dual-coil system because of the high defibrillation threshold.

Finally, high-energy shock delivery is also an indispensable factor in detection of lead failure. An R-wave synchronized shock will not induce VT/VF, making this method safer than normal DFT testing. However, it is possible to underestimate the diagnosis of short circuit because the shock is always delivered in systole. A conductor is not fixed in a lumen of the lead. It therefore may move like "sawing" action by the heart beat and also may be affected by posture and respiration,^12^ which could result in alternation of the distance from the conductor of the abraded area to the adjacent conductor, can, or coil. Thus, we emphasize that a single trial of the high-energy shock delivery will not guarantee lead integrity. If the shock fails to deliver, the lead is failing, but a correctly delivered shock cannot be interpreted with confidence. Multiple successful shock deliveries raise confidence, but the number of successful shocks needed for certainty is unknown and multiple shocks carry their own risks.

## Conclusion

We experienced a case of ICD shock delivery failure, which was likely caused by a short circuit in the Riata ST Optim lead. Exclusion of the SVC coil from the defibrillation system may be one of the measures to prevent a short circuit between the SVC coil and RV conductor.

**Key Teaching Points**

• The Riata ST Optim lead was developed by coating Optim over a Riata ST lead, which was recalled in 2011. Even though the rate of conductor externalization and longevity of the lead has improved, the risk of internal insulation breach following failure of shock delivery persists because Optim did not cover a layer between the superior vena cava (SVC) and right ventricular coil in the Riata ST Optim lead.
• Electronic short circuit is only evident when a high-energy current is delivered with shock therapy. It is difficult to predict failure by routine monitoring of shock lead impedance, even with automatic daily remote measurements.
• There are currently 3 possible approaches to prevent defibrillation failure, as follows: (1) excluding the SVC coil from the defibrillation system; (2) selecting an automatic shocking-vector adjustment algorithm (Dynamic Tx); and (3) conducting a high-energy shock delivery test.
